# Global longitudinal strain is an informative index of left ventricular performance in neonates receiving intensive care

**DOI:** 10.1038/s41598-024-59441-5

**Published:** 2024-04-17

**Authors:** Enrico Petoello, Alice Iride Flore, Silvia Nogara, Elena Bonafiglia, Maria Beatrice Lenzi, Olivia C. Arnone, Giovanni Benfari, Martina Ciarcià, Iuri Corsini, Koert De Waal, Leonardo Gottin, Benjamim Ficial

**Affiliations:** 1 Neonatal Intensive Care Unit, University and Hospital Trust of Verona, P.le A. Stefani 1, 37126, Verona, Italy; 2 Section of Cardiology, Department of Medicine, University of Verona, Verona, Italy; 3 Division of Neonatology, Careggi University Hospital of Florence, Florence, Italy; 4 Department of Neonatology, John Hunter Children’s Hospital, Newcastle, NSW Australia; 5 University of Newcastle, Newcastle, NSW Australia; 6 Intensive Care Unit, Department of Surgery, Dentistry, Maternity and Infant, University and Hospital Trust of Verona, Verona, Italy

**Keywords:** Ventricular Function, Left, Echocardiography, Global Longitudinal Strain, Intensive care, neonatal, Infant, Newborn, Neonatology, Cardiology

## Abstract

Echocardiographic assessment of left ventricular function is crucial in NICU. The study aimed to compare the accuracy and agreement of global longitudinal strain (GLS) with conventional measurements. Real-life echocardiograms of neonates receiving intensive care were retrospectively reviewed. Shortening fraction (SF), ejection fraction (EF) and S’ measurements were retrieved from health records. GLS was calculated offline from stored images. The association with stroke volume indexed for body weight (iSV) was evaluated by regression analysis. The diagnostic ability to identify uncompensated shock was assessed by ROC curve analysis. Cohen's κ was run to assess agreement. 334 echocardiograms of 155 neonates were evaluated. Mean ± SD gestational age and birth weight were 34.5 ± 4.1 weeks and 2264 ± 914 g, respectively. SF, EF, S’ and GLS were associated with iSV with R^2^ of 0.133, 0.332, 0.252 and 0.633, (all p < .001). Including all variables in a regression model, iSV prediction showed an adjusted R^2^ of 0.667, (p < .001). GLS explained 73% of the model variance. GLS showed a better ability to diagnose uncompensated shock (AUC 0.956) compared to EF, S’ and SF (AUC 0.757, 0.737 and 0.606, respectively). GLS showed a moderate agreement with EF (κ = .500, p < .001) and a limited agreement with S’ and SF (κ = .260, p < .001, κ = .242, p < .001). GLS was a more informative index of left ventricular performance, providing the rationale for a more extensive use of GLS at the cotside.

## Introduction

Neonatologist performed echocardiography (NPE) has been increasingly used to assess cardiac function in critically ill neonates, to early identify hemodynamic compromise, to understand the underlying pathophysiology, and to guide therapeutic management^1^. Current recommendations suggest interventions based on the quantification of left ventricle (LV) systolic function rather than on the qualitative assessment of myocardial function^2–4^. Several echocardiographic indices have been used to assess LV systolic performance in neonates requiring intensive care. Shortening fraction (SF) and ejection fraction (EF) quantify LV systolic function by measuring changes in dimension and volume, respectively^2^ Tissue Doppler imaging (TDI) allows quantification of myocardial velocity of a definite point of the heart. The peak systolic velocity (S’) of the movement of the myocardium at the lateral insertion of the mitral valve towards the apex has been conventionally adopted as a measurement of LV function. ^5^

However, these conventional measurements have numerous shortcomings. Firstly, SF and EF have limited accuracy in identifying myocardial dysfunction and significant variability within and between observers. Secondly, both these measurements are based on the assumption that the LV has a normal geometry, which is uncommon in neonates due to the increased pulmonary pressure, in particular during the transitional period^2,6,7^. Secondly, TDI measurements depend on the angle of insonation and do not allow for a distinction between passive and active motion of the heart ^5^. Thirdly, there is a lack of consensus on the reference ranges that should be used. The latter increases the variability between institutions^2^. Finally, although a multiparametric approach is recommended, there is no data on the relative contribution of each individual measurement to the diagnosis of LV systolic dysfunction. To the best of our knowledge, no diagnostic algorithm has been proposed so far to guide the clinician in a step-by-step process, with measurements and reference ranges, to evaluate LV systolic function in newborns.

To overcome the limitations of the conventional measurements of LV function, in the last decade, myocardial deformation imaging by 2D-speckle tracking echocardiography has been applied to assess the cardiac deformation (strain) during the cardiac cycle in three directions (longitudinal, circumferential and radial). This has been extensively evaluated in adults, where cardiac deformation in the longitudinal direction or global longitudinal strain (GLS) is considered a more sensitive index of LV systolic function compared to EF. GLS has better prognostic value in various cardiac diseases and better reproducibility. ^8^ There is a growing body of evidence that myocardial deformation imaging is feasible, reproducible, and provides enhanced diagnostic precision also in neonates^9–12^.

Our study aimed to compare the accuracy and the agreement between the conventional measurements of LV function and GLS in a large cohort of neonates. The accuracy was evaluated both using the stroke volume indexed for body weight as a comparator and testing the ability to identify neonates with uncompensated shock. Secondly, the agreement in classification as impaired vs. normal systolic function was evaluated.

## Materials and methods

### Study design

This was a single-center retrospective study conducted at a tertiary referral academic neonatal intensive care unit (NICU). Ethics approval was obtained from the local Research Ethics Committee. Parents or guardians of neonates gave their written informed consent. This was a convenience sample: all neonates, admitted from January to December 2022, who underwent echocardiographic assessment of LV function were included in the study. Exclusion criteria were: major congenital abnormalities, and congenital heart disease, except for patent ductus arteriosus (PDA) and patent foramen ovale.

During the study period, NPE was used to assess neonates with PDA, persistent pulmonary hypertension of the newborn (PPHN), shock and/or hypoperfusion, for the screening of congenital heart disease (CHD) and pulmonary hypertension in neonates with bronchopulmonary dysplasia (BPD) and research purposes. All examinations were performed using a Philips Epiq 7 system with 12 MHz probe (Philips Ultrasound, Andover, MA, USA). Images were acquired according to the recommendations of the American Society of Echocardiography^13^. Electrocardiograms were recorded simultaneously. Three cardiac cycles were digitally stored at high frame rate (100–130 Hz). Image quality was assessed according to Colan et al.^14^ Images considered poor or unusable were excluded from the analysis^15^.

### Conventional measurements of LV systolic function

**Figure 1:**
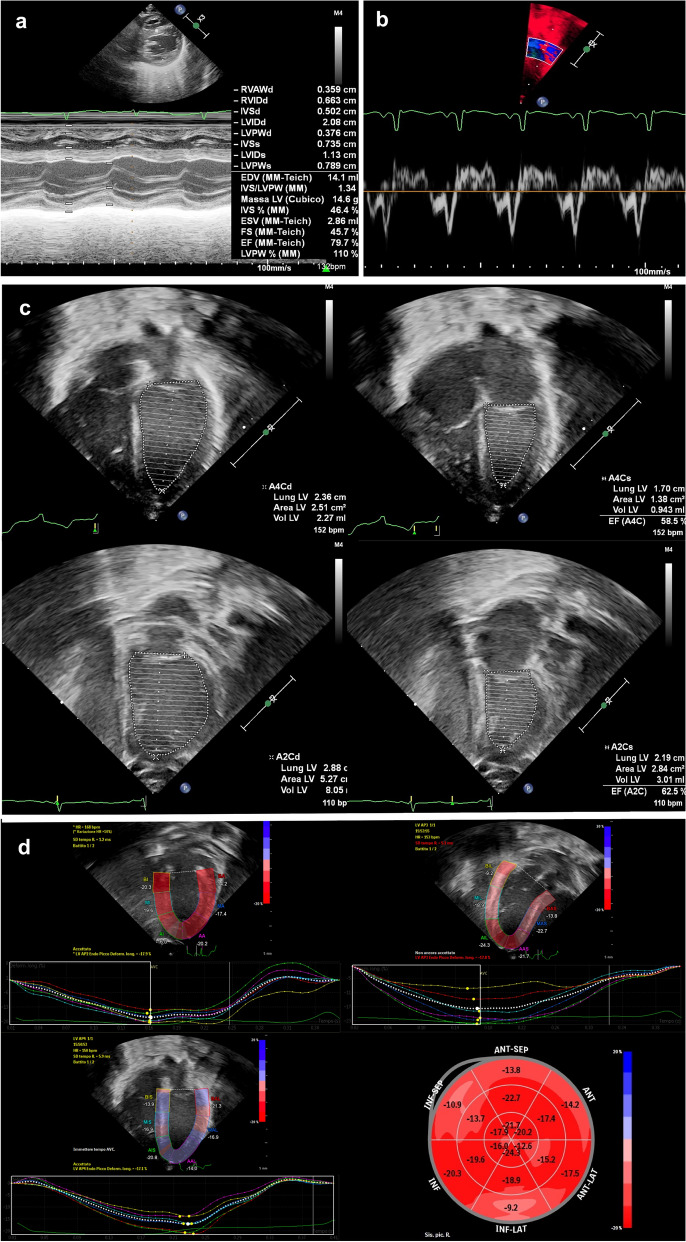
(**a**) M-mode image of a short axis view at the level just distal to the leaflet tips of the mitral valve showing shortening fraction (SF) calculation. (**b**) Tissue doppler imaging of an apical 4-chamber view with the sample volume at the lateral margin of the mitral valve, showing systolic peak velocity (S’) (**c**) Calculation of ejection fraction (EF) according to Simpson’s biplane method of disc. Apical 4-chamber views (top) showing the measurement of left ventricular end-diastolic (left) and end-systolic (right) volumes. Apical 2-chamber view (bottom) showing the measurement of left ventricular end-diastolic (left) and end-systolic volumes (right). (**d**) Longitudinal-Strain in 2-chamber view (top-left), 3-chamber view (top-right) and 4-chamber view (bottom-left). Bull’s-eye of global longitudinal strain (GLS). Bull’s eye plot derived from the longitudinal strain of the three apical views (bottom-right).

Conventional measurements of LV systolic function were retrieved from electronic health records. Measurements were done offline, immediately after scanning, using the QLAB software available on the ultrasound system. The following measurements were obtained as previously described: SF was calculated from M-mode images of a short axis view at the level just distal to the leaflet tips of the mitral valve (Fig. 1a)^2^ TDI was used to measure myocardial velocities from an apical 4-chamber view, placing the sample volume just apical to the lateral margin of the mitral valve: systolic peak velocity (S’) was measured (Fig. 1b)^5^ EF was calculated from a 4- and 2-chamber view based on the modified biplane Simpson’s method of disc (Fig. 1c)^6^

### Global Longitudinal Strain (GLS)

According to our echocardiographic protocol, an apical 4-, 3- and 2-chamber view with ECG trace was recorded, trying to obtain an optimal visualization of the LV wall and to avoid foreshortening of the LV. The analysis of digitally stored images was performed offline using the Philips QLAB software available on the ultrasound system, version 10.0 (https://www.usa.philips.com/healthcare/product/HCNOCTN145/qlab-cardiovascular-ultrasound-quantification-software#documents), by an operator blind to the conventional echo data, at least 4 weeks apart from image acquisition. As previously described, the QRS complex was used as a reference time-point. The region of interest (ROI), including six segments with different colors, was automatically traced by an endocardial point-and-click approach in each apical view (4-, 3- and 2-chamber view). The ROI was manually corrected, if needed, Then the software automatically tracked the movement of speckles to derive the deformation parameters. The 18-segment model was used; peak longitudinal strain from a 4-, 3- and 2-chamber view and GLS were calculated as previously reported (Fig. 1d)^16^.

### Stroke volume

Stroke volume (SV) was used to assess the accuracy of the measurements of LV systolic function as previously described in adults^17^. To calculate SV, aortic internal diameter was measured at the valve hinge points from a parasternal long axis view and aortic velocity time integral was measured from an apical 5-chamber view. SV was indexed for body weight (iSV)^18,19^.

### Statistical analysis

The neonates enrolled in the study were divided into two subgroups: neonates with impaired and normal LV function. The latter was defined as GLS ≤ -15^10,20^. Normality of data distribution was assessed by Shapiro–Wilk’s test. Data are presented as mean (± SD), median (interquartile range (IQR)) or count (%) for parametric, non-parametric and categorical variables, respectively. Differences between the two groups of neonates were evaluated using an independent-sample t-test, Mann–Whitney U test and χ^2^ test in case of parametric, non-parametric, and categorical variables, respectively. Pearson's partial correlation was run to assess the relationship between SF, EF, S’, GLS and iSV. Univariate and multivariate linear regression analysis was run to estimate the ability of the conventional LV systolic measurements (SF, EF, S’) and that of GLS to predict the iSV. Receiver-operating characteristic (ROC) curve analysis was used to assess the diagnostic ability of GLS, EF, S’ and SF to identify neonates with uncompensated shock. The latter was defined as blood pressure below the 3° centile according to gestational age and clinical signs of hypoperfusion (prolonged capillary refill time, tachycardia, tachypnea, mottled skin and/or cool extremities, reduced or absent urine output, lactic acidosis)^21^.

Cohen's κ was run to assess the agreement between GLS and the other conventional measurements (SF, EF and S’) on whether the left ventricular systolic function was judged impaired or normal. According to our institutional echocardiographic protocol, the following cut-off values to define impaired LV function were used as previously described: SF ≤ 25%, EF ≤ 55%, S’ ≤ 3 cm/s in neonates with gestational age (GA) < 30 weeks, S’ ≤ 3.5 cm/s in neonates with GA from 30 to 36 weeks, S’ ≤ 4.2 cm/s in neonates with GA ≥ 37 weeks. For the advanced echocardiographic imaging, we used a GLS > -15 to define an impaired LV function^5,16,22^. P value < 0.05 was considered significant. Data were analysed using SPSS 20 (SPSS, Chicago, Illinois, USA).

### Ethics approval

The study was approved by the Research Ethics committee of the University Hospital of Verona, no. 2132CESC. The parents or guardians of babies enrolled gave their written informed consent. All procedures performed in studies involving human participants were in accordance with the ethical standards of the institutional and/ or national research committee and with the 1964 Helsinki Declaration and its later amendments or comparable ethical standards.

## Results

From January to December 2022 a total of 460 echocardiograms of 215 neonates were performed and were assessed for eligibility. One hundred and ten echocardiograms of 44 neonates did not meet the inclusion criteria: in 39 echocardiograms a complete assessment of LV function was not performed, in 26 echocardiograms no stored images were found, lastly, 33 and 12 echocardiograms were excluded due to CHD and major congenital abnormalities, respectively.

Following the image quality assessment of the 4-, 3- and 2-chamber views of stored clips, 16 echocardiograms of 16 neonates were excluded. Finally, 334 echocardiograms of 155 neonates were included in the analysis. The study flow chart is shown in Supplemental Fig. 1.

The echocardiograms were performed for the following reasons: PDA assessment 108 (30.9%), screening CHD 101 (28.9%), screening BPD 39 (11.1%), shock and/or hypoperfusion 20 (5.7%); research purposes 78 (22.3%), PPHN 4 (1.1%).

### Clinical characteristics

The mean ± SD gestational age and birth weight of the 155 neonates included were 34.5 ± 4.1 weeks and 2264 ± 914 g, respectively, 73/155 were female (47.1%). The clinical characteristics at the time of echocardiography of the two groups of neonates with impaired and normal LV function are shown in Table 1.

**Table 1:** Clinical characteristics at the time of echo of the two cohorts of neonates with normal and impaired GLS.

	Impaired LV function (72 neonates)	Normal LV function (262 neonates)	P value
Gestational age (week)	34.4 ± 4.7	34.9 ± 4.1	0.36
Weight at birth (g)	2357 ± 1114	2289 ± 899	0.59
Weight at scan (g)	2348 ± 1101	2301 ± 858	0.70
Female	31 (43.1%)	113 (43.5%)	.95
Day of life	2 [1–2]	1 [1–2]	**0.02**
Ventilation			
None	38 (52.8%)	148 (56.5%)	0.66
Non-invasive ventilation	19 (26.4%)	102 (38.9%)	0.06
Invasive ventilation	15 (20.8%)	12 (4.6%)	** < 0.01**
FiO_2_	0.21 [0.21–0.23]	0.21[0.21–0.21]	0.67
iNO	9 (12.5%)	6 (2.3%)	**0.01**
Systolic blood pressure (mmHg)	66 ± 14	68 ± 9	0.52
Mean blood pressure (mmHg)	45 ± 11	47 ± 8	0.22
Diastolic blood pressure (mmHg)	37 ± 11	39 ± 9	0.44
Daily total fluid (ml/kg/die)	103 ± 42	94 ± 40	0.13
Vasopressor and/or inotrope	10 (13.9%)	4 (1.5%)	** < 0.01**
Sepsis at the time of echo	12 (16.7%)	25 (9.5%)	0.09
IUGR	4 (5.6%)	33 (12.6%)	0.13
Antenatal steroids	21 (29.2%)	53 (20.2%)	0.11

Values are presented as mean ± SD, median [IQR] and count (%).

*LV* left ventricle, *iNO* inhaled nitic oxide, *IUGR* intrauterine growth restriction.

Significant values are in bold.

### Echocardiographic assessment of LV function

Conventional echocardiographic measurements of LV systolic function and GLS are presented in Table 2. All echocardiographic parameters presented a statistically significant difference between the two groups of neonates with impaired and normal LV function: SF was 30.4 ± 8.6% and 35.0 ± 7.2% (p < 0.01), EF 50.6 ± 7.7% and 61.8 ± 6.4%, (p < 0.01), S’ 4.2 ± 0.9 and 5.0 ± 0.9 cm/s, (p < 0.01), iSV 0.9 ± 0.3 and 1.5 ± 0.4 ml/kg, (p < 0.01), GLS -11.9 ± 2.2% and -18 ± 2.1%, (p < 0.01), respectively.

**Table 2:** Echocardiographic parameters of LV systolic function of the two cohorts of neonates with normal and impaired LV function.

	Impaired LV function (72 neonates)	Normal LV function (262 neonates)	P value
SF (%)	30.4 ± 8.6	35.0 ± 7.2	** < .01**
Simpson’s biplane EF (%)	50.6 ± 7.7	61.8 ± 6.4	** < .01**
S’ (cm/s)	4.2 ± 0.9	5.0 ± 0.9	** < .01**
Indexed Stroke volume (ml/kg)	0.9 ± 0.3	1.5 ± 0.4	** < .01**
GLS (%)	–11.9 ± 2.2	–18 ± 2.1	** < .01**

Values are presented as mean ± SD.

*LV* left ventricle, *SF* shortening fraction, *EF* ejection fraction, *GLS* global longitudinal strain.

Significant values are in bold.

Pearson correlations matrix is shown in Supplemental Table 1. Small statistically significant correlations between S’ and SF (r = 0.28) and S’ and EF (r = 0.29) were found. Moderate statistically significant correlations between SF and EF (r = 0.33), SF and GLS (r =− 0.37), S’ and GLS (r =− 0.41), SF and iSV (r = 0.428), EF and iSV (r = 0.576) and S’ and iSV (r = 0.502) were found. Strong statistically significant correlations between EF and GLS (r =− 0.64) and GLS and iSV (r = − 0.796) were found.

### Univariate and multivariate regression analysis

**Figure 2:**
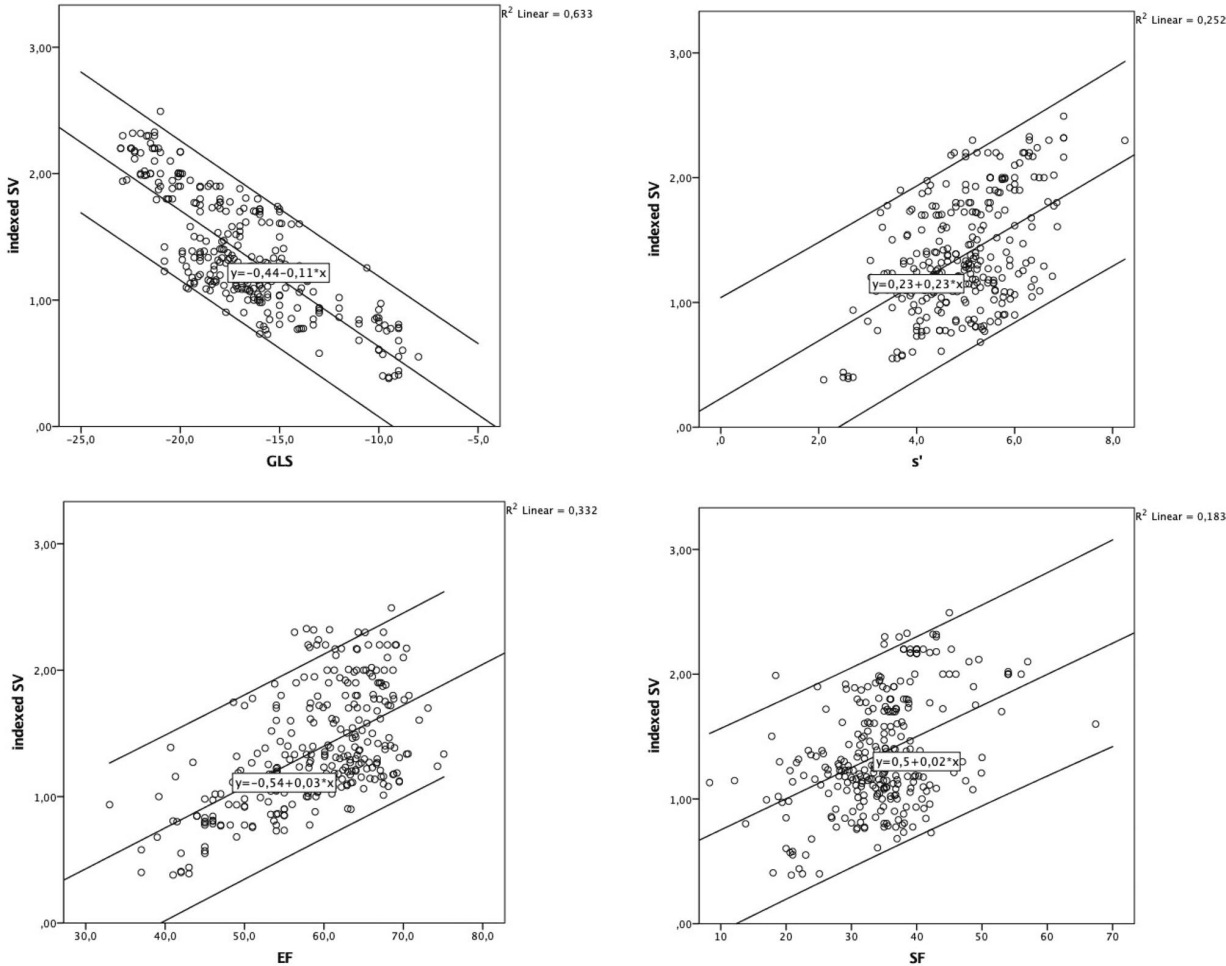
The scatterplots with the line of best fit, 95% CI and equation of SF, EF, S’, and GLS with the indexed stroke volume. SV  = *stroke volume*, EF  = *ejection fraction*, GLS  = *global longitudinal strain*, SF  = *shortening fraction.*

An univariate linear regression analysis was used to predict the indexed stroke volume based on SF, EF, S’ and GLS, respectively. The scatterplots with the line of best fit, 95% CI, and equation are shown in Fig. 2. SF, EF, S’ and GLS significantly predict iSV (all p < 0.001), with and R^2^ of 0.133, 0.332, 0.252, 0.633, respectively.

A multivariate regression analysis was run to predict iSV from SF, EF, S’, and GLS. There was no evidence of multicollinearity: the variance inflation factors of SF, EF, S’, and EF were 1.21, 1.69, 1.29, and 1.91, respectively. The multiple regression model statistically significantly predicted iSV, p < 0.001. R^2^ for the overall model was 0.672 with an adjusted R^2^ of 0.667. All four variables added statistically significantly to the prediction, p < 0.05. Results of the univariate and multivariate regression analysis are presented in Table 3.

**Table 3:** Univariate and multivariate linear regression analysis: Role of SF, EF, S’ and GLS on the indexed stroke volume (iSV).

Predictor	Univariate analysis					Multivariate analysis					
	B	95% CI		β	p	B	95% CI		β	p	% explained variance
		Lower limit	Upper Limit				Lower limit	Upper Limit			
SF	0.025	0.019	0.031	0.428	< .001	0.008	0.003	0.012	0.120	.002	8%
EF	0.032	0.027	0.038	0.576	< .001	0.006	0.000	0.011	0.097	.037	8%
S’	0.231	0.186	0.277	0.502	< .001	0.066	0.029	0.104	0.143	< .001	11%
GLS	− 0.108	− 0.117	− 0.98	− 0.796	< .001	0.087	− 0.101	- 0.073	− 0.621	< .001	73%

*B* unstandardized regression coefficient, *CI* confidence interval, *β* standardized coefficient, *SF*, shortening fraction, EF ejection fraction, *GLS* global longitudinal strain.

### ROC curve analysis

In Supplemental Fig. 2 we present the ROC curve of GLS, EF, S’, and SF for the diagnosis of uncompensated shock. The AUC (95% CI) of GLS, EF, S’, and SF was 0.956 (0.902–1.000), 0.757 (0.625–0.888), 0.737 (0.634–0.840) and 0.606 (0.422–0.789), respectively. The DeLong test showed that the AUC of GLS was superior to the AUC of SF, EF and S’ (p < 0.02).

### Agreement between conventional measurements and GLS

A moderate agreement was found between GLS and EF: κ = 0.500, p < 0.001. A limited agreement was found between GLS and S’ and between GLS and SF: κ = 0.260, p < 0.001 and κ = 0.242, p < 0.001, respectively.

## Discussion

Monitoring the adequacy of the oxygen delivery to cells and tissues and applying the appropriate therapeutic interventions is a dogma of intensive care medicine. Longitudinal monitoring of SV, which is the amount of blood ejected through the aortic valve during systole, is of paramount importance. SV, together with heart rate and arterial oxygen content, is a determinant of oxygen delivery ^3^. As the summative event of the cardiac cycle, from the perspective of the critical care physician, SV is the most pragmatic indicator of the well-being of the heart and the cardiovascular system, independent of EF^19,23^.

In this study, the association with iSV of GLS and the other conventional measurements of LV function, their ability to identify neonates with uncompensated shock, and their agreement were evaluated for the first time in real-life echocardiograms of neonates requiring intensive care.

Firstly, a strong association of iSV with GLS was found, in contrast to the weaker associations with SF, EF and S’. Secondly, when GLS and all the conventional parameters were included in the regression model to predict iSV, more than 70% of the variance of iSV was explained by GLS, with only a limited contribution from S’, EF and SF. Thirdly, the ability of GLS to identify neonates with uncompensated shock was superior compared to the other measurements of LV function. Finally, a limited agreement was found between GLS and the conventional echocardiographic measures of systolic function.

Our data confirmed that SF, EF and S’ are far from being accurate in the neonatal population, showing only a moderate correlation with iSV and a limited ability to identify neonates with uncompensated shock.

SF was the measure that showed the lowest accuracy. It relies on normal LV septal morphology, the latter being commonly altered in neonates, due to the increased pulmonary pressure after birth. Moreover, previous findings showed that the longitudinal shortening of the LV is the major contributor to the SV^17^. SF, which assesses the radial shortening, is of little value. We feel that its use, as a single parameter of LV function, should be discouraged in neonates, although the available guidelines still include SF in the quantitative assessment of LV function^2,3^.

The accuracy of both S’ and EF was slightly superior but still suboptimal. S’ evaluates the myocardial velocities during the cardiac cycle only in one specific region of the heart, adjacent to the mitral annulus, it is angle-dependent and cannot differentiate active from passive motion^5^.

EF calculation with Simpson’s biplane method of disc is based on geometric assumptions, which may lead to inaccuracies in measurements, especially in dilated ventricles or in the presence of abnormal septal motion^24^. Although EF is a cornerstone in the assessment of LV systolic function and the management of heart failure, there is growing evidence suggesting that EF is not a sensitive marker of myocardial dysfunction. EF, which measures changes in the volume of the LV cavity, is only an indirect measure of LV function, and is prone to geometrical confounders, specifically in the case of LV hypertrophy or reduced LV cavity^25^.

Our data underlines that, from the neonatologist’s perspective, there is an urgent need for more accurate measures of LV systolic function, due to the poor accuracy of the conventional measures and their limited agreement with GLS. We believe that advanced echocardiographic imaging is a promising tool. GLS provides a direct measure of myocardial systolic function^26^. We acknowledge that the inter-vendor variations and the need for off-line analysis can inhibit the adoption of GLS in clinical practice. However, future technical developments and the availability of automated analysis may facilitate this process. In the current study, GLS was calculated using the software available on the ultrasound system, as in the real-world assessment of LV function at the cotside, showing an optimal potential for the clinical application of this technique.

GLS showed a strong association with iSV at the univariable analysis and was the major determinant of the variance of iSV, compared to the conventional measurements, at the multivariate analysis. We hypothesized that GLS is a more sensitive index of LV dysfunction compared to EF due to the complex myocardial architecture with three distinct layers of fibers with different orientations^27^. GLS allows a better assessment of the deformation of the longitudinal sub-endocardial fibers, which are more vulnerable to wall stress and mostly contribute to stroke volume^17^. Moreover, it overcomes the shortcomings of the conventional measurements: it does not rely on geometrical assumption, it is not angle-dependent, it differentiates between active and passive motion of the heart, and it detects impaired myocardial function even in the presence of LV hypertrophy and reduced LV volume^25^.

In adults and older children, there is growing evidence of the prognostic and diagnostic superiority of GLS over conventional parameters in a wide range of diseases, with optimal reproducibility. Previous data confirmed that myocardial deformation imaging is feasible, reproducible and accurate also in neonates with PDA, PPHN, hypoxic ischemic encephalopathy, and congenital diaphragmatic hernia^16,28–31^. Our findings provide evidence that GLS should be added to the armamentarium of the NPE.

### Limitations

This study has several limitations. Firstly, we assessed the accuracy against iSV measured by echo. Given the fact that thermodilution is not feasible in the neonatal population and other techniques (e.g. arterial pulse contour analysis, bioimpedance and bioreactance) lack accuracy, the gold standard for the evaluation of SV in children is the cardiovascular magnetic resonance.

However, the latter is costly, time-consuming and not easily available in the NICU^32^. We previously showed in a smaller cohort of stable neonates that SV measured by echo is a robust measure, with optimal reproducibility and strong agreement with cardiovascular magnetic resonance^18,33^. Therefore, we believe that SV measured by echo is the only comparator, available at the cotside, in such a large cohort of neonates.

Secondly, this was a retrospective analysis of echocardiograms performed by different physicians. Due to the study design, we did not take into account the interobserver variability, which can be substantial particularly for EF and SF, as previously reported^8^. However, the hemodynamic team in our center routinely follows protocols of image acquisition and analysis to uniform measurements and minimize variability between and within observers.

Thirdly, we did not assess the intra and interobserver reproducibility of the off-line analysis of GLS and of the other measurements of LV function. However, we recently presented prospectively collected data on reproducibility, which confirmed an optimal reproducibility both within and between observers for GLS, SF, EF and S’^12^.

## Conclusions

Using real-life echocardiograms from a large cohort of neonates requiring intensive care we showed that GLS was a more informative index of LV performance compared to the conventional measures (EF, SF, S’). Our findings provide the pathophysiological and clinical rationale for a more extensive use of GLS in the quantitative assessment of LV systolic function, to guide management. Further studies are needed to evaluate the association with relevant outcomes and the efficacy of therapeutic interventions, before widespread incorporation of GLS into mainstream clinical practice.

### Supplementary Information

Supplementary Information.

## Data Availability

The data that support the findings of this study are available from the corresponding author upon reasonable request.

## Abbreviations

BPD: Bronchopulmonary dysplasia
CHD: Congenital heart disease
EF: Ejection fraction
GA: Gestational age
GLS: Global longitudinal strain
IQR: Interquartile range
iSV: Indexed stroke volume
LV: Left ventricle
NICU: Neonatal intensive care unit
NPE: Neonatologist performed echocardiography
PDA: Patent ductus arteriosus
PPHN: Persistent pulmonary hypertension of the newborn
ROI: Region of interest
ROC: Receiver-operating characteristic
SF: Shortening fraction
SV: Stroke volume
TDI: Tissue doppler imaging
