# Supplementary material for: Global longitudinal strain is an informative index of left ventricular performance in neonates receiving intensive care
Source: Sci Rep. 2024 Apr 17;14:8881. doi: 10.1038/s41598-024-59441-5 (PMC11024117; doi:10.1038/s41598-024-59441-5)
Supplement: Supplementary file 1 — Supplementary Information. [file 41598_2024_59441_MOESM1_ESM.docx]

**Global longitudinal strain is an informative index of left ventricular performance in neonates receiving intensive care.**

Enrico Petoello, MD,^1^ Alice Iride Flore, MD^1^, Silvia Nogara, MD^1^, Elena Bonafiglia, MD^1^, Maria Beatrice Lenzi, MD^1^, Olivia C. Arnone, MD^1^, Giovanni Benfari, MD, PhD^2^, Martina Ciarcià, MD^1^, Iuri Corsini, MD^3^, Koert De Waal, MD, PhD^4,5^, Leonardo Gottin, MD^6^, Benjamim Ficial, MD^1^*

^1^ Neonatal Intensive Care Unit, University and Hospital Trust of Verona, Verona, Italy

^2^ Section of Cardiology, Department of Medicine, University of Verona, Verona, Italy

^3^ Division of Neonatology, Careggi University Hospital of Florence, Florence Italy

^4^ John Hunter Children’s Hospital, department of neonatology, Newcastle NSW, Australia

^5^ University of Newcastle, Newcastle NSW, Australia

^6^ Intensive Care Unit, Department of Surgery, Dentistry, Maternity and Infant, University and Hospital Trust of Verona, Verona, Italy

*Corresponding author: Benjamim Ficial, ORCID 0000-0001-6056-5757,

Neonatal Intensive Care Unit, Azienda Ospedaliera Universitaria Integrata Verona, P. le A. Stefani 1, 37126, Verona, Italy.

phone: +390458123106; fax: +390458123373; email: benjamim.ficial@gmail.com

**Supplemental Fig. 1**

Study flow chart.

*Abbreviations: LV = left ventricle, CHD = congenital heart disease*

**Supplemental Fig. 2**

ROC curve analysis of the diagnostic ability of GLS; EF, s’ and SF to identify neonates with uncompensated shock.

*Abbreviations: EF = ejection fraction; GLS = global longitudinal strain; SF = shortening fraction.*

**Supplemental Table 1**

**Pearson correlations between echocardiographic parameters of LV systolic function**

|  | SF | EF | s’ | GLS | iSV |
| --- | --- | --- | --- | --- | --- |
| SF | 1 |  |  |  |  |
| EF | 0.33** | 1 |  |  |  |
| s’ | 0.28** | 0.29** | 1 |  |  |
| GLS | - 0.37** | - 0.64** | - 0.41** | 1 |  |
| iSV | 0.428** | 0.576** | 0.502** | - 0.796** | 1 |

*Abbreviations: SF, shortening fraction; EF, ejection fraction; GLS, global longitudinal strain; iSV, indexed stroke volume.*

*** p < .01*
